# Residency redeployment during a pandemic: Lessons for balancing service and learning

**DOI:** 10.36834/cmej.70267

**Published:** 2020-09-23

**Authors:** Fernanda Claudio, Armand Aalamian, Beth-Ann Cummings, Mathew Hannouche, Patrizia Zanelli, Leon Tourian

**Affiliations:** 1 McGill University, Quebec, Canada

## Introduction

The COVID-19 pandemic has required a rapid healthcare system response worldwide, including in Canada.^1^ As of July 21 2020, 111,124 Canadians were diagnosed with COVID-19 with 8858 deaths, an estimated case fatality rate of 8%.^2^ In Quebec there are a total of 57,796 cases, with the Greater Montreal area the epicenter ^3^. To respond to health service delivery needs while maintaining standards of physician training, the Postgraduate Medical Education (PGME) Office of McGill University’s Faculty of Medicine developed a resident redeployment strategy to support its teaching hospitals.

Redeployment was framed as an integral aspect of doctors’ implicit social contract.^4^ McGill’s redeployment strategy took place between April 3 and June 30. During that time, 376 residents from 47 training programs were redeployed primarily to intensive care units and newly-established COVID-19 wards. The PGME Office requires that residents’ standing contracts be respected, including supervision and assessment. A pandemic redeployment assignment, of one- to two-weeks duration, provided structure to the learning experience. Recognizing the ongoing need for resident redeployment, a survey was developed and administered with a continuous quality improvement lens. We investigated two themes: 1) residents’ experiences of redeployment, and 2) the impact of redeployment on resident education.

Pandemics suggest unique learning opportunities in medical knowledge, clinical skills, teamwork and resilience. Few studies investigate educational impact on trainees’ participation in emergency response. We gathered preliminary ‘lessons learned,’ aiming to discern satisfaction and effectiveness of COVID-19 redeployment learning activities, and frame a larger study of educational outcomes of resident pandemic redeployment.

## Methods

A 13-question, tailor-made survey was developed, consisting of pre-selected response categories and free-text fields, and made available to 1200 residents in 70 specialties across MUHC, using the Microsoft Forms platform. Questions included: whether a redeployment rotation was completed; type of service attended two weeks prior to redeployment; scholarly activities; type of COVID-19 assignment; site of redeployment; level of satisfaction with redeployment; reasons for level of satisfaction; preferences for redeployment scheduling; satisfaction with communication; and suggestions for improvements.

We collated numerical results and analyzed these using Microsoft Forms. Free-text results were thematically analyzed by comparing and contrasting codes attributed to particular words.

This article was internally reviewed by Dr. Peter Nugus of the Institute of Health Sciences Education, McGill University. The survey was conducted by the Postgraduate Medical Education office as a Quality Improvement exercise. No IRB was necessary.

## Results

One hundred forty six residents responded, of whom 79 were redeployed. Fifty eight were redeployed to COVID wards, ten to COVID-ICU units, four to ICUs, three to Internal Medicine wards, and four to other sites (including Clinical Teaching Units). Most residents worked at one of three major teaching hospitals (76). Most expressed satisfaction (49) noting exceptional faculty support, alignment between competence and work, and reasonable workload. Residents who reported dissatisfaction (20) had concerns about inadequate personal protective equipment (PPE), heavy workload, short notice to deploy and poor orientation, and perceived gaps between knowledge and needs of COVID clinical settings and their training. One resident reported aiding dying patients overnight as emotionally difficult.

Recommendations for improvement emerging from free-text responses are categorized as: 1) longer notice for preparation and improved communication within sites; 2) suitable PPE (availability and fitting); 3) appropriate context of redeployment (ie. better calibration of level of competence to service, including allocation to familiar settings); and 4) preparation for emotional and stress-inducing demands of palliation.

## Summary

Our study aimed to understand residents’ experiences of COVID-19 redeployment and impacts on educational experience. This is an important foundation for our next phase – for which we invite collaborations with colleagues engaged in similar emergency educational activities elsewhere – to understand relationships between educational experiences from the pandemic and particular specialties-to inform educational design. Preliminary lessons learned include: 1) deployment to high stress and complex environments requires emotional and technical preparation; and 2) supportive faculty in familiar surroundings enhances the learning experience in high-stress services. The pandemic response underscores the person-centred, collegial and communal support that must always underpin technical learning.
